# Population-Level Persistence of Immunity 2 Years After the PsA-TT Mass-Vaccination Campaign in Mali

**DOI:** 10.1093/cid/civ602

**Published:** 2015-11-09

**Authors:** Nicole E. Basta, Ray Borrow, Abdoulaye Berthe, Awa Traoré Eps Dembélé, Uma Onwuchekwa, Kelly Townsend, Rahamatou M. Boukary, Lesley Mabey, Helen Findlow, Xilian Bai, Samba O. Sow

**Affiliations:** 1 Division of Epidemiology and Community Health, University of Minnesota, Minneapolis; 2 Fogarty International Center, National Institutes of Health, Bethesda, Maryland; 3 Vaccine Evaluation Unit, Public Health England, Manchester Royal Infirmary, United Kingdom; 4 Centre pour le Développement des Vaccins, Ministère de la Santé, Bamako, Mali

**Keywords:** meningococcal vaccines, immunity, seroprevalence, Africa

## Abstract

***Background.*** In 2010, Africa's first preventive meningococcal mass vaccination campaign was launched using a newly developed *Neisseria meningitidis* group A (NmA) polysaccharide–tetanus toxoid conjugate vaccine, PsA-TT (MenAfriVac), designed specifically for the meningitis belt. Given PsA-TT's recent introduction, the duration of protection against meningococcal group A is unknown.

***Methods.*** We conducted a household-based, age-stratified seroprevalence survey in Bamako, Mali, in 2012, 2 years after the vaccination campaign targeted all 1- to 29-year-olds. Randomly selected participants who had been eligible for PsA-TT provided a blood sample and responded to a questionnaire. Sera were analyzed to assess NmA-specific serum bactericidal antibody titers using rabbit complement (rSBA) and NmA-specific immunoglobulin G (IgG) by enzyme-linked immunosorbent assay. The proportion of participants putatively protected and the age group- and sex-specific rSBA geometric mean titers (GMTs) and IgG geometric mean concentrations (GMCs) were determined.

***Results.*** Two years postvaccination, nearly all of the 800 participants (99.0%; 95% confidence interval [CI], 98.3%–99.7%) maintained NmA-specific rSBA titers ≥8, the accepted threshold for protection; 98.6% (95% CI, 97.8%–99.4%) had titers ≥128, and 89.5% (95% CI, 87.4%–91.6%) had titers ≥1024. The rSBA GMTs were significantly higher in females than in males aged <18 years at vaccination (*P* < .0001). NmA-specific IgG levels ≥2 µg/mL were found in 88.5% (95% CI, 86.3%–90.7%) of participants.

***Conclusions.*** Two years after PsA-TT introduction, a very high proportion of the population targeted for vaccination maintains high antibody titers against NmA. Assessing the duration of protection provided by PsA-TT is a priority for implementing evidence-based vaccination strategies. Representative, population-based seroprevalence studies complement clinical trials and provide this key evidence.

Efforts to prevent the large-scale outbreaks of meningococcal disease that have led to significant morbidity and mortality in the African meningitis belt, a region stretching from Senegal to Ethiopia, changed dramatically in 2010 when a newly developed group A meningococcal polysaccharide–tetanus toxoid protein conjugate vaccine, PsA-TT or MenAfriVac, was introduced in the first-ever preventive mass vaccination campaign in Burkina Faso, Mali, and Niger [1–4]. Developed by the Meningitis Vaccine Project, a partnership between the World Health Organization (WHO) and PATH with funding from the Bill & Melinda Gates Foundation, PsA-TT transformed the reactive vaccination strategy in place in the Meningitis Belt since the 1970s, which utilized polysaccharide meningococcal vaccines to respond to meningococcal disease outbreaks, into a preventive campaign that is implementing mass vaccination of individuals aged 1–29 years in >25 countries in Africa [5]. Evidence has indicated that PsA-TT is highly immunogenic; the vaccine was licensed based on immunogenicity alone [2, 6–8]. By the end of 2014, >217 million people in 15 countries had been vaccinated with PsA-TT [9]. The introduction of PsA-TT has dramatically altered the epidemiology of meningococcal disease in Africa, and significant reductions in the incidence of meningococcal A disease [10, 11] and meningococcal A carriage [12, 13] have been achieved in countries where the vaccine has been introduced.

As with all recently developed and newly introduced vaccines, questions remain about the duration of protection provided by PsA-TT. Assessing the duration of protection has been identified as a priority for the prevention and control of meningitis in Africa [14]. Evaluating antibody persistence both in the context of clinical trials and in population-based epidemiologic studies is an important component of investigating the impact conjugate meningococcal A vaccination has had on immunity in this region. Meningococcal conjugate vaccines including PsA-TT join together polysaccharides from the outer membrane of *Neisseria meningitidis* to a protein carrier such as tetanus toxoid. Conjugate vaccines elicit a more robust immune response than polysaccharide-only vaccines, which are limited in that immunity wanes after 3–5 years and in that they are not immunogenic in very young children [15–17]. Although conjugate meningococcal C and ACWY vaccines provide a longer duration of immunity than comparable polysaccharide vaccines, antibody levels have been shown to wane rapidly following vaccination [18, 19]. Booster doses have been added to vaccination schedules in both the United States, where meningococcal ACWY conjugate vaccine is recommended for 11- and 12-year-olds with a booster dose at age 16 years [20], and the United Kingdom, where meningococcal C conjugate vaccine is recommended for infants and booster doses are given at 12 months and around 14 years [21]. Booster doses for these meningococcal conjugate vaccines have been recommended due to concerns about waning immunity during periods of high risk [18, 22–24], further emphasizing the need to understand the duration of immunity following PsA-TT introduction has been introduced in areas where meningococcal disease is highly endemic.

To assess the duration of protection and changes in population-level immunity over time following the 2010 introduction of PsA-TT, we established a cohort in 2012 among residents of Bamako, Mali. Here we report the results of the first seroprevalence survey undertaken in the cohort, 2 years after the PsA-TT mass vaccination campaign.

## METHODS

### Study Design

This study was conducted in Mali, a country hyperendemic for meningococcal disease located in the meningitis belt. Mali reported >35 000 suspected cases of meningitis to the World Health Organization over the past 2 decades [25, 26]. Participants were recruited from the Banconi district of Bamako, the capital city of Mali, where >130 000 residents live, as part of the US National Institutes of Health–funded PsA-TT (MenAfriVac) Antibody Persistence (MAP) study. Launched in 2012, the MAP study aims to assess changes in population-level immunity following the 2010 PsA-TT mass vaccination campaign. The design has been briefly described previously [27]. Participants were randomly selected from an existing demographic surveillance system maintained by the Center for Vaccine Development-Mali (CVD-Mali) using a household-based, age-stratified sampling design. Randomly selected participants were eligible for the study if they were aged 1–29 years at the time of the 2010 vaccination campaign, were living in Banconi during the campaign and at the time of the study, had not participated in any of the PsA-TT clinical trials, and were healthy enough to provide a blood sample. Participants aged ≥18 years were asked to provide written consent. Participants aged 13–17 years were asked to provide written assent, and a parent or guardian was asked to provide written consent. Younger children provided oral assent and a parent or guardian provided written consent. Participants enrolled in the study in December 2012 were asked to provide up to 8.5 mL of blood and to respond to a questionnaire.

### Immunologic Assessment

Blood samples collected in plastic gold-top vacutainer tubes containing clot activator (Becton, Dickinson and Company catalog no. 367953) were immediately inverted 4–6 times and then stood upright to clot for at least 30–45 minutes at room temperature before being stored in a cool box (2°C –8°C) for transport. Samples were transported to the laboratory within 4–6 hours where they were centrifuged at 4000 revolutions per minute for 15 minutes. The serum was extracted, divided into aliquots, and stored in cryovials at −80°C prior to shipment on dry ice to the Vaccine Evaluation Unit, Public Health England (Manchester, United Kingdom). Sera were analyzed to assess complement-mediated serum bactericidal antibody (rSBA) levels using baby rabbit complement as an exogenous source. The *N. meningitidis* (NmA) reference strain used was F8238 (A:P1.20,9). rSBA titers were determined by a standard protocol, as previously described [28], and results were given as the reciprocal of the final dilution of sera that resulted in 50% killing of colonies after 60 minutes. Sera were also analyzed to assess NmA-specific immunoglobulin G (IgG) concentrations by enzyme-linked immunosorbent assay (ELISA) [29].

### Statistical Analysis

Data were managed and analyzed using Stata SE software, version 13.1 (Stata Corp, LP). Participants were stratified into 5 age groups during the 2012 survey to ensure representation from all ages eligible to receive PsA-TT during the 2010 mass vaccination campaign. Results are reported (unless otherwise noted) by age at survey, 2 years after vaccination: 3–4 years, 5–7 years, 8–12 years, 13–19 years, and 20–31 years. The age-specific proportion of participants with rSBA titers greater than or equal to the standard threshold of 8 was calculated along with the 95% confidence intervals (CIs). This threshold was established initially for evaluation of meningococcal C vaccines [19, 30, 31] and has been applied to both quadrivalent ACWY and single-group A meningococcal vaccines, which have been licensed based on their immunogenicity alone without evaluation of direct efficacy against disease [8, 32–34]. The proportion protected (and 95% CI) with titers ≥128 and ≥1024 were also determined as a more conservative assessment of immune persistence, which has been advocated previously [31, 33]. rSBA titers below the lower limit of quantification (LLOQ) of 4 were assigned a value of 2. The proportion of participants with NmA-specific IgG concentrations ≥2 µg/mL was calculated along with the 95% CI. IgG concentrations below the LLOQ of 0.19 µg/mL were assigned a value of 0.095 µg/mL. The rSBA geometric mean titers (GMTs) and IgG geometric mean concentrations (GMCs) were determined by calculating the geometric mean and 95% CIs. To assess sex differences in GMTs and GMCs by age, the values were log base 2 and log base 10 transformed, respectively, and assessed using *t* tests allowing for unequal variance. The differences in proportions putatively protected were assessed using 2-sample tests of proportions. The Pearson correlation coefficient was used to assess the correlation between the log-transformed rSBA titers and IgG concentrations.

### Ethics Approval

Ethical approval for this research was granted by the Princeton University Institutional Review Board, the University of Minnesota Institutional Review Board, and the University of Bamako Ethics Committee.

## RESULTS

### Participant Characteristics

Eight hundred participants were enrolled in the seroprevalence survey between 10 December 2012 and 10 January 2013. Key demographic characteristics of the participants have been reported previously [27]. In brief, 57.1% of participants were female; 49.0% reported their ethnicity as Bambara, 10.9% Sarakolé, 10.0% Malinke (all part of the Mande ethnic group), and 11.6% Peuhl (Fulani); 39% lived in a household where at least 1 resident smoked, although only 1.3% reported that they themselves smoked; and, of those aged >18 at the time of the survey, 35.4% had never attended school. According to the enrollment criteria, all were eligible to receive PsA-TT during the December 2010 campaign; 99.5 (95% CI, 99.0%–100.0%) reported receiving PsA-TT. The 4 individuals who reported that they did not receive the vaccine are included in the following analyses because our aim was to assess antibody persistence in the population 2 years after MenAfriVac introduction among a representative sample of residents targeted for vaccination. In the Banconi district, part of Commune 1 in Bamako, an estimated 91% (95% CI, 88.9%–91.1%) of eligible residents was vaccinated in 2010, according to a survey conducted 1 month after the campaign [35].

### Serum Bactericidal Antibody Results

**Figure 1.**
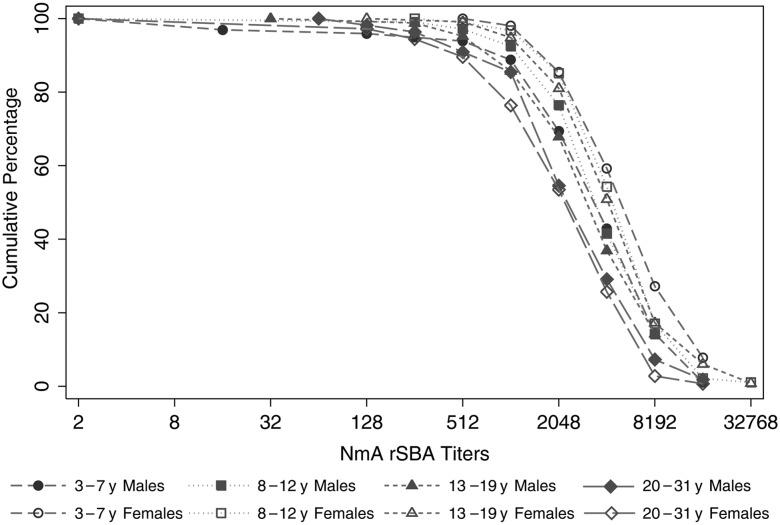
Reverse cumulative distribution of *Neisseria meningitidis* (NmA)–specific serum bactericidal antibody titers using rabbit complement (rSBA) by age group and sex 2 years after PsA-TT vaccine introduction in Bamako, Mali.

The rSBA titers ranged from undetectable levels (8 participants, all of whom reported being vaccinated) to 32 768 (2 participants, 1 of whom reported not having been vaccinated). Two years after the introduction of PsA-TT, 99.0% (95% CI, 98.3%–99.7%) of participants had rSBA titers ≥8, 98.6% (95% CI, 97.8%–99.4%) had titers ≥128, and 89.5% (95% CI, 87.4%–91.6%) had titers ≥1024. Figure 1 shows the reverse cumulative distribution of rSBA titers stratified by age group and sex; nearly all participants had rSBA titers well beyond the putative threshold of protection across all strata. Averaging across all age groups, the rSBA GMT was 2157 (95% CI, 1988–2341). High GMTs were observed across all age groups for both males and females (Table 1), with the highest GMTs among females aged 5–7 years (3603 [95% CI, 3035–4277]) and 3–4 years (3183 [95% CI, 2050–4944]) at the time of the survey. Interestingly, among those <18 years old at the time of vaccination, females had significantly higher rSBA GMTs than males overall (3112 [95% CI, 2831–3414] vs 2033 [95% CI, 1760–2349], respectively, *P* < .0001) and by age group (Table 1).

**Table 1.** Comparison of Serum Bactericidal Antibody Using Rabbit Complement Geometric Mean Titers and Immunoglobulin G Geometric Mean Concentrations by Age Group and Sex Among a Randomly Selected, Representative Sample of Residents of Bamako, Mali, 2 Years After the Introduction of PsA-TT

Age at Vaccination, y	Age at Survey, y	Sex	No.	MenA rSBA GMT (95% CI)	*P* Value	MenA IgG GMC (95% CI), µg/mL	*P* Value
1–2	3–4	M	28	1448 (722–2904)	.055	1.5 (1.1–2.1)	.49
		F	22	3183 (2050–4944)		1.9 (1.2–3.0)	
3–5	5–7	M	70	2008 (1411–2857)	.004	4.5 (3.4–6.2)	.29
		F	81	3603 (3035–4277)		5.6 (4.3–7.3)	
6–10	8–12	M	106	2274 (1857–2784)	.033	11.6 (9.3–14.5)	.90
		F	94	3005 (2566–3520)		11.4 (9.4–13.8)	
11–17	13–19	M	84	1998 (1604–2489)	.009	17.1 (13.1–22.2)	.033
		F	116	2879 (2449–3384)		24.3 (20.1–29.3)	
18–29	20–31	M	55	1592 (1200–2111)	.16	19.4 (13.0–29.0)	.57
		F	144	1230 (971–1557)		17.1 (14.0–20.8)	

Abbreviations: CI, confidence interval; GMC, geometric mean concentration; GMT, geometric mean titer; IgG, immunoglobulin G; MenA, group A meningococcus; rSBA, serum bactericidal antibody titers using rabbit complement.

### IgG ELISA Results

**Figure 2.**
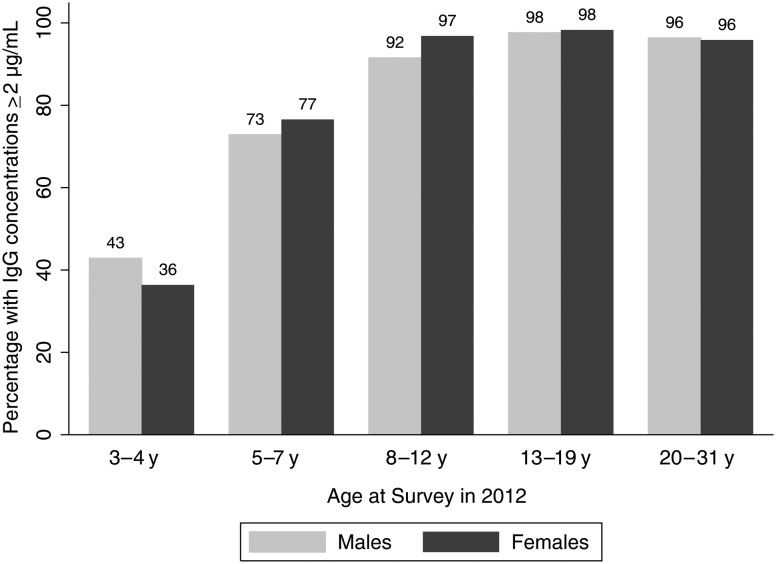
Percentage of participants with *Neisseria meningitidis* (NmA)–specific immunoglobulin G (IgG) concentrations ≥2 µg/mL by age group and sex 2 years after PsA-TT vaccine introduction in Bamako, Mali.

NmA-specific IgG concentrations ranged from undetectable levels (1 participant) to >750 µg/mL (1 participant). The IgG GMC across all age and sex groups was 11.3 µg/mL (95% CI, 10.3–12.4 µg/mL). Overall, 88.5% (95% CI, 86.3%–90.7%) of participants had concentrations ≥2.0 µg/mL (Figure 2). IgG GMCs were highest among those aged 11–17 years and 18–29 years at the time of vaccination (Table 1). There was no statistically significant difference in IgG GMCs between males and females within age strata except for those aged 11–17 years at the time of vaccination (Table 1). There was some evidence of a very weak correlation between the rSBA titer and IgG concentrations (*r* = 0.10, *P* = .005) (data not shown).

## DISCUSSION

We provide clear evidence that high levels of NmA-specific rSBA and IgG antibodies persist 2 years after the mass vaccination campaign ended among residents of Bamako, Mali, eligible for PsA-TT vaccination. This study was designed to assess changes in immunity over time in a cohort of individuals who were eligible for the 2010 mass vaccination campaign. The results reported here are from December 2012, at the time that the cohort was established. Participants provided blood samples again in 2014 (3.5 years post–vaccine introduction) and will be followed up in the future to further investigate long-term changes in population-level immunity after the introduction of this new meningococcal group A vaccine in a resource-poor country hyperendemic for meningococcal disease.

Population-based epidemiological studies are uniquely suited to complement clinical trials of new vaccines, can aid in the investigation of the duration of protection, and are especially informative when newly developed vaccines are evaluated and licensed on the basis of immunogenicity alone, as in the case of PsA-TT. In comparison to PsA-TT clinical trial results, our results indicate that the level of antibody persistence exceeds baseline levels pre–vaccine introduction in countries of the African meningitis belt even though prevaccination antibody levels have been found to be high in this region [36, 37]. In particular, a PsA-TT trial conducted among 2- to 29-year-olds in Mali, The Gambia, and Senegal found prevaccination rSBA GMTs of 223.3 (95% CI, 181.3–274.9), which increased to 4712.6 (95% CI, 4336.0–5122.0) 4 weeks after PsA-TT vaccination [8]. Our results suggest that although the rSBA GMTs from our representative sample are lower than the peak in titers seen 4 weeks after vaccination, the GMT of 2157 (95% CI, 1988–2341) observed 2 years after vaccine introduction is significantly higher than the GMT observed among trial participants prior to vaccination. This suggests that while antibody levels have declined somewhat following vaccine introduction, they have not declined to prevaccination levels. Sow et al [8] reported NmA-specific IgG GMCs of 2.1 µg/mL (95% CI, 1.9–2.5) prevaccination, which increased to 65.6 µg/mL (95% CI, 60.0–71.6) 4 weeks after vaccination in trial participants aged 2–29 years. Our results suggest some waning of IgG following an initial peak in antibody response, which may also be a result of reduced exposure to NmA carrier strains and fewer opportunities to develop immunity in the 2 years since widespread PsA-TT introduction. In addition, prior to PsA-TT vaccination in Burkina Faso, a seroprevalence survey was undertaken to establish baseline rSBA GMTs and NmA-specific IgG GMCs [38]. Our results from Mali again indicate higher antibody levels 2 years after vaccine introduction than observed prior to the launch of mass vaccination in the African meningitis belt.

Interestingly, we also found a significant difference between persisting antibody levels between males and females, with females vaccinated before the age of 18 years having higher rSBA GMTs than males. Whereas evidence indicates that females exhibit stronger antibody responses than males following vaccination with influenza, BCG, measles, mumps, and rubella, and other vaccines [39], we are not aware of any studies of PsA-TT or other meningococcal vaccines that have investigated sex differences in antibody persistence following vaccination. Little is known about the biological mechanisms that lead to sex differences in immunity, although hypotheses regarding the role of sex hormones, the microbiome, and genetic factors have been investigated [39]. We believe this relationship should be explored further to determine if these differences are clinically relevant and whether the mechanisms that drive these differences can be elucidated.

We aimed to assess immunity against group A meningococcal disease at the population level by investigating antibody levels among a large, representative sample of individuals who were eligible for PsA-TT during the December 2010 mass vaccination campaign in Mali. However, because we did not have access to prevaccination sera samples from participants, we were not able to assess the kinetics of immunity among vaccinated individuals over time; rather, we report on age- and sex-specific patterns of immunity in the population. While PsA-TT vaccination rates in Bamako were reportedly high [35], vaccination status was self-reported in this study. We took several steps to ensure that our findings that high antibody responses have persisted among those targeted for vaccination during the PsA-TT campaign were robust to limited information about individual-level vaccination status. First, our enrollment criteria required participants to have been residing in Bamako during the time of the mass vaccination campaign and to have been eligible for vaccination. These 2 criteria were verified at the time participants were randomly selected using an established demographic surveillance system. Second, we included all participants in the analyses, regardless of self-reported vaccination status or the ability to produce their PsA-TT vaccination card at the time of the survey. This decision was made to ensure that our estimates were conservative. Our conclusion that immunity has persisted in the population applies to the underlying population from which the sample was drawn, namely, those eligible and targeted for vaccination during the mass vaccination campaign regardless of individual-level vaccination status. Our study benefits from a large, representative sample and a standardized protocol for assessing antibody levels that allows valid comparison with PsA-TT clinical trial results, which were also produced by the same laboratory. Furthermore, our results are consistent with clinical trial results and evidence from epidemiologic studies that have found significant reduction in disease incidence 2 years after PsA-TT introduction in Chad [10, 11] and significantly lower rates of NmA carriage 2 years after PsA-TT vaccination in Burkina Faso [12, 13]. The results of our study contribute to this evidence and add an important population-based perspective on changes in immunity over time following PsA-TT introduction. Future assessment of the duration of protection provided by PsA-TT and other new vaccines should include population-based epidemiological seroprevalence studies. The results reported here are based on the initial survey of a newly established cohort. Longer-term assessment of changes in NmA-specific immunity is under way and will provide key insight about whether booster doses may be needed to maintain the dramatic decline in incidence following PsA-TT introduction in the African meningitis belt.
